# Transmission of Acquired Characteristics

**Published:** 1908-02-08

**Authors:** 


### Transmission of Acquired Characteristics.

This problem, which is at present occupying the attention of most biologists, should be of interest to the medical profession as well. In fact, it might almost be said that the members of the profession have greater facility for the study of the question than the professed evolutionist. Statistics, though unfortunately a dangerous weapon in the hand of the sophist, may be nevertheless of great value in the determination of approximate results. Medical men, especially students in charge of out-patients, invariably come in contact with hundreds of cases, the observation of which, if considered from a definite point of view, would lead to profitable results.

Take the question of the inheritance of disease. It should be possible for the intelligent student to question his patients as to their antecedents, and, in at least a proportion of cases, to obtain coherent and reliable answers. Such an inquiry should not be carried on in a slipshod, perfunctory fashion. The highest accuracy must be attained, and when sufficient cases have been observed, the results must be carefully analysed and classified. The inheritance of malformations is another problem of equal interest to mankind at large. It will rest largely with medical students of this and succeeding generations whether it will be possible to formulate a law of heredity on which scientific treatment can be based. The work of Francis Galton ought to be a sufficient pattern to follow.

It seems too early almost to consider the application of Mendel's theory to man, though evidence might be analysed with a view to its substantiation. Those who are at present working out this theory necessarily feel that they are the pioneers in a very doubtful land. Mendel showed, in the case of peas, that on crossing two kinds the first generation of offspring resembled one parent. On the self-fertilisation of this generation the resulting offspring resembled the original pair in the proportion of three to one. He therefore regarded one parent as "dominant" in character and the other as "recessive." Two-thirds, however, of the dominants of the second generation produced offspring in exactly the same proportion—namely, three dominants to one recessive, and this result was obtained throughout when two-thirds of these dominants were fertilised. The recessive always bred true, as also did one-third of the dominants.

This brief outline may serve to indicate the nature of the problem of inheritance. It tends to show that the offspring do not necessarily progress, but rather return, to the original parental type. If this theory could be applied to men, we should be a step further in the matter of social reform, which is so much desired nowadays. Modern Socialists think that they see in economic redistribution the panacea for all evil, but it will rather rest with the scientist and the medical man to decide whether the race is capable of amelioration, and whether the offspring of certain marriages will not be as evil as their parents before them, however satisfactory their environment. The Turks have a proverb to this effect, which is both interesting and instructive: "The son of a wolf will be a wolf, even if he grow up with a man." Considerations, therefore, of a truly scientific nature should give pause to those who, with a somewhat crude emotionalism, rush headlong for reform. Great improvements are so seldom produced by "fulguration," and the problem of economic and hygienic betterment can only be handled by scientists. It is the medical man who can lead the van in this campaign.

All great work in science has been the outcome of indefatigable observation and unflagging accuracy. The greater the number of workers, the greater the chance of success. It is to be hoped, therefore, that all those who have such vast opportunities for helping the progress of science will not foolishly let them go. Let them take as their motto *Carpe diem*, which, being interpreted, means, make your daily criticism and observation.

